# Pericardiectomy for constrictive pericarditis: single-center experience in China

**DOI:** 10.1186/s13019-015-0237-6

**Published:** 2015-03-19

**Authors:** Peng Zhu, Mingjie Mai, Ruobin Wu, Cong Lu, Ruixin Fan, Shaoyi Zheng

**Affiliations:** 1 Department of Cardiovascular Surgery, Southern Medical University, GuangZhou, People's Republic of China; 2 Department of Cardiovascular surgery, Guangdong General Hospital, Guangdong Academy of Medical Sciences, Guangzhou, People's Republic of China

**Keywords:** Constrictive pericarditis, Pericardiectomy, Surgery, Prognosis

## Abstract

**Objective:**

Pericardiectomy is associated with a high prevalence of morbidity and mortality. We evaluated the predictors of in-hospital complications and outcome for pericardiectomy procedure for patients with constrictive pericarditis (CP) in a single-center in China.

**Methods:**

One-hundred sixty-five patients who underwent pericardiectomy for CP between January 1990 and December 2012 at our hospital were evaluated.

**Results:**

The mean age of the study cohort was 36.79 ± 18.52 years. The approach was through a median sternotomy in 91.5% of patients. Cardiopulmonary bypass was used in 14.5% (24/165 patients). Unadjusted rates of mortality and complication were approximately 5.4% and 23%, respectively. The main cause of death was severe low cardiac output syndrome. Major complications were postoperative low cardiac output syndrome, reoperation for bleeding, pneumonia, mediastinitis, chylothorax and cerebral infarction. One-year survival was 92%. One-year follow-up revealed that New York Heart Association functional class III or IV, age, intraoperative use of cardiac pulmonary bypass and hemodialysis were associated with increased mortality and morbidity.

**Conclusions:**

Total pericardiectomy is associated with lower perioperative and late mortality, and the extent of pericardial resection should be decided according to individual conditions. Perioperative management and complete release of the thickened pericardium of the left ventricle should prevent postoperative complications.

## Background

Constrictive pericarditis (CP) usually presents as a result of chronic fibrous pericardial thickening and calcification of the pericardium [1]. The thickened and fibrotic pericardium causes reduced cardiac output as a consequence of impaired diastolic filling of the cardiac chambers [2].

In the past, the main cause of CP was tuberculosis (TB). The etiology of chronic CP has changed over the past few decades, and TB is no longer the predominant cause [3,4]. The etiology of pericarditis is quite diverse and most patients progress to symptoms severe enough to require surgical intervention.

Pericardiectomy is the accepted treatment for improving cardiac hemodynamics in CP, but isassociated with a high prevalence of morbidity and mortality [3-6]. The risk factors associated with pericardiectomy have been reported, but the information is limited (especially for procedures conducted in China) [7]. The outcomes of pericardiectomy would greatly benefit from appropriate surgical strategies and perioperative medical management based on the identification of perioperative risk factors [6-9].

Here, we highlight the predictors of morbidity and mortality of pericardiectomy based on our experience of improving the management of CP. For this purpose, short-term surgical outcomes after pericardiectomy for CP were investigated in a single center.

## Patients and methods

We carried out a retrospective analysis of patients who underwent surgery for CP with associated cardiac diseases at our hospital. Between January 1990 and December 2012, we identified 165 patients diagnosed with CP.

The diagnosis of CP was made on the basis of clinical, echocardiography, cardiac catheterization, surgical, and pathological criteria. The most important diagnostic tool is the suspicion of CP in a patient with signs and symptoms of right-sided heart failure that are disproportionate to pulmonary of left-sided heart disease. Typical symptoms and signs are a prominent change in the x and y descent in jugular venous pulse, dyspnea upon exertion, palpitations, abdominal distension, as well as edema in the ankles or legs. Echocardiography revealed a severely thickened or calcified pericardium and cardiac catheterization revealed elevated end-diastolic pressure and the “square root sign” of right ventricular pressure tracing. Surgical and pathological findings were reviewed to confirm the preoperative diagnosis.

Between January 1990 and December 2012, 165 consecutive patients (108 males) underwent pericardiectomy for CP. Table 1 lists the causes of CP in this series. Heavy smokers had to cease smoking 1 week before surgery. A TB received isoniazid, rifampicin, or ethambutol for ≥9 months after surgery.

**Table 1:** **Relationship between constrictive pericarditis and the underlying etiology**

Etiologic factors	No	%
Indeterminate	106	64
Tuberculosis	39	23
Post-surgery	19	11
Chest trauma	1	1
Infection	1	1

Among the 165 patients with CP, 39 patients had a history of pulmonary TB, but none had TB of the bones or joints. The baseline clinical details of the patients are summarized in Table 2. A total of 119 patients had exertional dyspnea, 113 patients had distended jugular veins, 111 patients showed lower-limb edema, 92 patients had abdominal symptoms, and 55 patients suffered from palpitations. When classifying patients using the preoperative functional class set by the New York Heart Association (NYHA), 144 patients (88%) were in class III/IV. The median duration of symptoms before pericardiectomy was 19 months (range, 1-7 years).

**Table 2:** **Descriptive data of properativeclincial details**

Variable*	Result
Age, y	36.79 ± 18.52
Male sex	108(65%)
Diabetes	2(1%)
NYHA functional class	
II	21(12%)
III or IV	95(57%)
IV	49(29%)
Renal dysfunction	2(1%)
Cardiac symptom and signs	
Exertional dyspnea	119(72%)
Raised jugular vein distension	113(68%)
Lower limb edema	111(67%)
Abdominal distension	92(65%)
Palpitation	55(33%)
Smoking history	69(42%)
Current smoking	15(9%)
Hypoalbuminemia	68(41%)
Pleural effusion	98(59%)
Pulmonary infiltrates	86(52%)
Pericardial calcification	81(48%)
Heart valve disorder	
Aortic regurgitation	2(1%)
Mitral regurgitation	16(10%)
Tricuspid regurgitation	17(10%)
Arrhythmia	95(58%)
Previous cardiac operation	19(12%)
History of myocardial infarction	3(2%)

*Qualitative variables are presented as number(%) and results of continuous variables as mean ± standard deviation; Hypoalbuminemia defined as a serum albumin concentration of less than 34 g/L, was 21% at the time of admission in adult hospitalized patients.

NYHA = New York Heart Association.

Initial laboratory assessments showed hypoalbuminemia in 68 patients. Chest radiography revealed pleural effusions (n = 98), pulmonary infiltrates (n = 86), and pericardial calcification (n = 63). Electrocardiography revealed non-specific T-wave changes in 47 patients, atrial fibrillations in 36 patients, and premature ventricular contraction in 12 patients. Echocardiography revealed a thickened pericardium in 142 patients. Preoperative computed tomography (CT) or magnetic resonance imaging (MRI) was done in all patients, and part of the pericardium was demonstrated to be thickened or calcified.

One-hundred and fifty-one subjects underwent surgery through a median sternotomy, and pericardiectomy was done through a left antrolateral thoracotomy in the remaining 14 patients. Constricting layers of epicardium were removed when possible. The pericardium was palpated to identify a relatively soft and uncalcified area after median sternotomy, and the thymus removed laterally. An #-shaped incision was made over the pericardium. Dissection was started at the base of the aorta, extended downwards to the lateral and posterior walls of the left ventricle and the pulmonary veins, followed by the diaphragmatic pericardium. The pericardium over the right atrium and venae cavae was resected last. The myocardium was then exposed, to achieve mobilization of the heart down to and beyond the phrenic nerves. If calcified plaques penetrating the epicardium were present, we left small “islands” of calcified pericardial tissue. Cardiopulmonary bypass (CPB) was avoided during surgery, except for: four patients needing removal calcified spicules; five patients requiring concomitant replacement of the mitral valve; nine patients needing concomitant tricuspid repair; one patient needing removal of a thrombus in the left atrium; one patient requiring concomitant replacement of the aortic valve; and three patients needing coronary artery bypass due to severe stenosis in the coronary arteries in patients with prior myocardial infarction(Table 2).

Left anterolateral thoracotomy through the fifth intercostal space was conducted in 14 patients. The decision for the approach was dependent on the personal preference of the operating surgeon. Left anterolateral thoracotomy offers excellent exposure of the anterolateral and inferior aspects of the left ventricle, with minimal manipulation and retraction of the left phrenic nerve. Carrying out decortications across the sternum and onto the right atrium or venae cavae during surgery is difficult. Hence, increasing number of surgeons prefer a midline incision and median sternotomy.

Surgical findings included a markedly thickened pericardium, calcification (108/165), caseous necrosis in the pericardial cavity (66/165)and a calcified pericardium penetrating the myocardium (36/165). The preoperative mean central venous pressure was 22.3 ± 5.9 mmHg and reduced to 9.8 ± 4.2 mm-Hg after surgery (p < 0.001). Surgical pathology specimens were examined for granulomas (58/165), as well as inflammation and fibrin deposition (97/165) . All bacterial cultures was negative.

### Statistical analyses

Results are the mean ± standard deviation or percentages. For risk analyses, a multiple logistic regression model was developed using a forward stepwise variable selection method. The Fisher exact test or χ^2^ test was used for categorical variables. Student’s *t-*test was employed for continuous covariates. P values ≤0.05 was considered significant. Analyses were undertaken using SPSS version 20.0 (SPSS, Chicago, IL,USA).

## Results

All deaths were cardiac-related. They occurred in the perioperative period as a result of low cardiac output syndrome due to right-heart failure. The 30-day mortality was 5.4% (9/165). Morbidity was due to low-cardiac-output syndrome (n = 17), acute renal failure (n = 5), respiratory insufficiency (n = 4), mediastinitis (n = 4), re-exploration for bleeding (n = 4) and acute stroke (n = 1) (Table 3). A total of 129 patients had significant improvement in their NYHA status and 25patients had a better outcome before discharge from hospital. After discharge from hospital, one patient was re-hospitalized to undergo redo surgery for recurrent CP, at 10 months of follow-up. Two patients were lost to follow-up after 6 months. One-year survival in 154 patients was 93.3%.

**Table 3:** **Intra-operative and postoperative information for pericardiectomy**

Variable*	Result (%)
Intraoperative information:	
Thoracotomy	14(8.4)
Median sternotomy	151(91.5)
Cardiopulmonary bypass	24(15)
Concurrent operation	
Mitral repair or replacement	5(3)
Aortic replacement	1(0.6)
Tricuspid repair	9(5.4)
Coronary Artery Bypass Grafting	3(1.8)
Left atrial thrombus	1(0.6)
Atrial septal defect repair	1(0.6)
CVP change, mmHg	
Preoperative CVP	22.3 ± 5.9
Postoperative CVP	9.8 ± 4.2
Postoperative complications	
Reopration for bleeding	4(2.4)
Low output syndrome	17(10.3)
Renal failure	5(3)
Mediastinitis	4(2.4)
Pneumonia	4(2.4)
Stroke	1(0.6)
Chylothorax	2(1.2)
Intra-Aortic balloon pump	3(1.8)
Intensive care unit stay, days	2.5 ± 1.7
Hospital stay, days	32 ± 13.9
In-hospital mortality	9(5.4)

*Qualitative variables are presented as number(%) and results of continuous variables as mean ± standard deviation.

CVP = central venous pressure.

The multivariate analysis of composite surgical mortality or major morbidity are summarized in Table 4. Multiple analysis showed that preoperative NYHA function (odds ratio (OR), 3.78; p < 0.001), age (OR,1.08; p = 0.02 ), CPB during surgery (OR, 2.13; p = 0.038) and hemodialysis (OR, 20.24; p < 0.001) after surgery were each positively associated with increased mortality and morbidity (Table 4).

**Table 4:** **Multivariate predictors of composite surgical mortality and major morbidity in 165 patients**

Covariate	Odds ratio	P value
	(95% confidence interval)	
Age	1.08(1.04-1.12)	0.02
Male sex	0.67(0.29-1.47)	0.64
Diabetes	0.94(0.26-3.45)	0.92
NYHA	3.78(1.77-8.98)	<0.001
Surgical approach (thoractomy/sternotomy)	1.36(0.26-8.87)	0.725
Intraoperative use of CPB	2.13(1.03-4.57)	0.038
Hemodialysis	20.24(6.78-58.53)	<0.001

NYHA = New York Heart Association; CPB = cardiopulmonary bypass.

## Discussion

The etiology of CP has varied between series during the past few decades. Idiopathic or viral pericarditis is the predominant cause in the western world, but TB is still a common cause of CP in “developing” and “underdeveloped” countries (especially in Asia) [2,7,9,10]. Patients with radiation-induced heart disease (RIHD) were not observed in our study, and 23% patients have a history of lung TB. The mean age of 36 years in this series is about 10-years younger than that reported in earlier studies [6-8]. This finding reflects the difference in the etiology of CP.

CP tends to be more common in males, and our study was consistent with previous studies [5-9]. There is no obvious explanation for this finding, but previous series have found the male-to-female preponderances for CP to vary between 1.7:1 and 4:1. There were two-times as many males as females in our series, and, interestingly, we found that being female gender was associated with lower morbidity (less than half).

One obstacle to successful treatment of CP is diagnostic uncertainty. In health, the pericardium is ≈ 3-mm thick. Traditionally, (especially due to TB), pericardial constriction has been associated with a normal-sized heart with restricted diastolic filling secondary to a rigid calcific pericardial shell that has a thickness of >6 mm. Today, diffuse calcification and thickening of the pericardium occurs much less commonly in patients with RIHD or after open-heart surgery. Thus, patients have signs and symptoms of right-sided heart failure that are disproportionate to left ventricular dysfunction or valve disease [11]. The challenge is to determine whether abnormalities are caused by pericardial restraint, myocardial restriction, or both.

No single approach should be used to diagnose all cases of CP [12]. The diagnostic approach should be individualized for patients. The primary diagnosis varies, depending upon to whom patients were first referred. The prominent signs are pleural effusions, ascites, leg edema, and increased jugular venous pressure. Sometimes, however, patients may not present with sufficient signs or symptoms for a definitive diagnosis to be made. In such cases, additional imaging is required and recommended.

Echocardiography can provide important information for the diagnosis of CP and for its differentiation from restrictive cardiomyopathy, and should be the initial non-invasive imaging employed [9-13]. Compared with echocardiography, CT and MRI have proved good and reliable investigations that are available widely. They are recommended for patients whose symptoms and signs suggest the possibility of pericardial constriction. Minimal pericardial calcifications and fibrocalcific thickened layers are early detectable using thoracal CT and MRI. CT or MRI was carried out in all our patients and demonstrated calcification or thickening of the pericardium in 152 patients. Careful history-taking and physical examinations, as well as the appropriate use of Doppler echocardiography and high-resolution CT or MRI, may aid in an early diagnosis.

Surgical management remains the only effective treatment for this potentially curable disorder [6-10]. In our experience, pericardiectomy should at least be considered if or before a patient develops NYHA functional class- III heart failure. In our center, pericardiectomy is carried out within 1 week after the diagnosis, before clinical manifestations become worse. Various approaches and methods(left anterolateral thoracotomy; median sternotomy; U incision with the base of the U lying at the left sterna border; bilateral thoracotomy) have been described since Rehn and Sauerbruch conducted a successful pericardial resection for the chronic CP [4]. The most commonly used approach is median sternotomy (especially if pericardiectomy is carried out routinely using CPB). The choice of approach appears is based on the personal preference of the surgeon.

Median sternotomy provides more radical clearance of the pericardium over the right atrium and venae cavae, and allows extensive pericardial removal using CPB[6,13]. In the present study, of all patients with pericardial thickening ≥3 mm, 14 (8.4%) underwent left anterolateral thoracotomy, and 151 (91.5%) underwent median sternotomy. CPB aids in surgical dissection by emptying the ventricular cavities to define clearly the appropriate plane of dissection, and facilitates the management of inadvertent cardiac injury. In the event of inadvertent excessive bleeding (n = 4), patients (n = 3) could be connected easily to CPB under median sternotomy. The disadvantage of using CPB is the potential for increased bleeding and other related complications. Chowdhury and colleagues suggested that delayed improvement and persistent symptoms are most commonly the result of incomplete decortications [9]. However, Schwefer et al suggested that long-term outcome is related not only to extent of the surgery but also the etiology of pericardial disease and preoperative NYHA status [12]. In the present study, despite total pericardiectomy, nine patients had early postoperative deaths due to low-output syndrome. Autopsy findings suggested that myocardial fibrosis and atrophy in these patients.

A correlation between NYHA class and overall or early survival has been observed by our research team and others scholars, and is the basis for advocating early pericardiectomy [14]. In this series, low-output syndrome, and reoperation for bleeding and renal failure were the most common postoperative complications. Improved perioperative management and medical therapy are important to avoid low cardiac output and restoration of right-heart function. Diuretics, inotropes, and vasodilators are the best medical therapies in the perioperative period [10]. However, there is a temptation, if faced with a much raised venous pressure and edema, for overzealous use of diuretics. Such management can lead to sudden death due to electromechanical dissociation, as we have observed. Intra-aortic balloon pumps and hemodialysis are advisable for patients with an advanced NYHA functional class.

In this series, the diagnosis of TB in CP was almost always confirmed by pathological means. Tuberculous CP can involve significant involvement of the lungs [2]. Preoperative chronic lung disease also has considerable negative effect on surgical results. Effective interventions can include aggressive physiotherapy, fiberoptic bronchoscopy along with aggressive screening for postoperative pneumonia. Blood transfusion may be needed for CP patients suffering from TB due to long-term malnutrition and deprivation of cardiogenic nutrients [7]. In general, 9-month anti-TB therapy for patients with suspected TB-based CP should be started after pericardiectomy.

### Limitations

Complete percardiectomy may not achieve total restitution in some patients suffering from CP a long period before surgery. Long-term follow-up data on survival are lacking, which limits our ability to understand the long-term benefits of complete percardiectomy. Radiation-induced pericarditis was has been reported to be a predictor of worse outcome, but we do not know if this was the cause in our patients. The only information regarding the etiology of CP in the present study was medical history and pathology.

Patients with preoperative hemodynamic cardiac catheterization were excluded from our cohort. Hence, there was increased difficulty in differentiating between constriction and restrictive cardiomyopathy. We could not adjust for surgical-team or surgeon-specific characteristics that could have made the analysis more robust.

## Conclusions

Pericardiectomy was associated with a lower prevalence of mortality, and postoperative low-output syndrome, as well as early normalization of hemodynamics. Careful perioperative management and surgical intervention upon or before right-heart failure may improve outcome. This is accomplished more readily through median sternotomy in patients with CP. Routine use of CPB during pericardiectomy is not necessary.

## Abbreviation

CP: Constrictive pericarditis
TB: Tuberculosis
RIHD: Radiation-induced heart disease
CPB: Cardiopulmonary bypass
CVP: Central venous pressure
NYHA: New York heart association
CT: Computer tomography
MRI: Magnatic resonance imaging
